# Syncope in Aortic Stenosis: Not Always What It Seems

**DOI:** 10.7759/cureus.36716

**Published:** 2023-03-26

**Authors:** Akshay Maharaj, Joel Teelucksingh

**Affiliations:** 1 Internal Medicine, Port of Spain General Hospital, Chaguanas, TTO; 2 Internal Medicine, San Fernando General Hospital, San Fernando, TTO

**Keywords:** arrythmia, vomiting, vomit, postprandial hypotension, post prandial hypotension, hyperorality, rest, syncope, aortic stenosis

## Abstract

Aortic stenosis is a common valvular pathology and may also have atypical presentations outside the classic triad of chest pain, syncope, and shortness of breath. Some patients may not present with the symptoms of the triad. This patient instead presented with syncope and vomiting. Statistically, the most common cause of syncope at rest in the setting of aortic stenosis is due to an arrhythmia rather than the valve itself limiting cardiac output. In a comorbid Alzheimer's patient who has developed hyperorality, postprandial hypotension can also result in syncope at rest. Therefore, syncope at rest should raise alarm for nonvalvular etiologies such as arrhythmia. This case study also aims to establish an association between syncope at rest and hyperorality in the setting of aortic stenosis, a first study so far. Additionally, it highlights an unusual presentation of aortic stenosis where syncope occurs at rest associated with vomit in the absence of chest pain or shortness of breath.

## Introduction

Aortic stenosis is a common valvular disease with a prevalence of 29% in people aged 65 and above [1]. Moreover, up to 9% of individuals aged 75 and higher have severe aortic stenosis. Aortic stenosis typically presents with the triad of chest pain upon exertion, shortness of breath, and syncope. However, in some cases, there may be exceptions to this triad such as in patients with diabetes where chest pain may be absent. Furthermore, lesser common manifestations of this disease may include vomiting and nausea.

In stenosis, the aortic valve calcifies and resists the forward flow of blood which raises left ventricular afterload, thereby resulting in a relatively fixed cardiac output. Thus, in such a condition that causes systemic hypotension, the heart is unable to augment cardiac output as per the demands of the body, which can lead to cerebral hypoperfusion and syncope. This is classically seen in exercise sessions, where beta 2 receptors induce skeletal muscle vasodilation which lowers blood pressure. Normally, baroreceptors are involved in detecting this change and compensate by increasing heart rate and stroke volume. However, aortic stenosis prevents patients from meeting this increased exertional demand. This can result in chest pain at rest and elevated cardiac biomarkers without coexistent coronary artery disease. This highlights that the presence of an arrhythmia such as atrial fibrillation can result in alterations from the natural exertional symptoms [2].

Valvular stenosis can result in pressure and volume overload of the left ventricle and atria. The presence of atrial dilatation greatly increases the risk of atrial fibrillation. Atrial fibrillation has a prevalence of 2-17% in mild-moderate aortic stenosis but a higher prevalence of 16-51% in severe aortic stenosis [3]. Additionally, aortic stenosis was commonly identified in 2-5% of patients with atrial fibrillation [4]. Hence, it can be stated that valvular defects are closely associated with arrhythmia.

Apart from atrial fibrillation, other arrhythmias may occur in aortic stenosis. In studies that analyzed ventricular arrhythmia in symptomatic severe aortic stenosis before transcatheter aortic valve implantation via 24-hour Holter monitoring, pre-ventricular complexes were present in 48% and non-sustained ventricular tachycardia in 9-29% [5,6].

There is a paucity of literature on the relationship between it and syncope at rest in the setting of aortic stenosis. This may lead to misinterpretation that the valve is the underlying cause of the syncope. This case report will further delve into this and demonstrate how the patient’s history can indicate a non-valvular cause of syncope. If this underlying cause is not addressed, the patient may continue to experience syncope even after the valve is repaired.

## Case presentation

This is the case of an 87-year-old male of East Indian descent. He had a known history of Alzheimer's disease, type 2 diabetes, and coronary artery disease. His medications included metformin 500mg per os (PO) twice daily, sitagliptin 50mg PO once daily, and gliclazide 60mg PO once daily. He also took aspirin 81mg PO once daily, rosuvastatin 20mg PO once daily, furosemide 20mg PO once daily, tamsulosin 0.4mg PO at night, dutasteride 5mg PO once daily, and nitroglycerin as needed.

He initially presented with a syncopal episode and vomiting after climbing a flight of stairs in June 2021. He was diagnosed with unstable angina of the anterolateral wall. Orthostatic hypotension was ruled out as an etiology. A follow-up with an echocardiogram showed he had moderate-severe aortic stenosis and moderate mitral regurgitation with a dilated left atrium. The echocardiogram also showed an ejection fraction between 50-55% and the peak/mean gradient across the valve was 51.1/34.4 mmHg. ECG showed sinus rhythm with first-degree atrioventricular (AV) block, ventricular rate of 80bpm, QTc 456ms, QRS 121ms, PR interval 306ms, and left axis deviation/ hypertrophy. Carotid Doppler ultrasonography also showed 0.7cm by 0.1cm non-stenotic plaque on the right common carotid. There was no sonographic evidence of hemodynamically significant stenosis. Since June 2021, the patient experienced chronic headaches, generalized weakness, and malaise, which progressively worsened with time. The patient declined workup that involved invasive procedures such as trans-catheter balloon valvuloplasty or coronary angiography.

In May 2022, he developed another syncopal episode but this time at rest. He also developed hyperorality, which involved eating more frequent meals. The family described him as ‘never being full’. Furthermore, he also developed irritability and his memory impairment worsened. He experienced numerous syncopal episodes from May to September, 2022, which progressively became more frequent until it was occurring every day. Each episode lasted for 10-15 minutes and was accompanied by urinary incontinence and was consistently followed by vomiting. Some episodes were accompanied by slurred speech and facial dropping which resolved. Of note, the syncope almost exclusively occurred in the 5-7 pm interval after eating and at rest. The family noted his systolic blood pressure would drop to 80-90 mmHg around this time. There was no hypoglycemia recorded during these syncopal episodes.

On September 20, 2022, he had three consecutive syncopal episodes. These episodes were accompanied by agonal breathing and a weak pulse. There was a period of apnea lasting for approximately one minute. Following the return of consciousness, the patient reported total vision loss, which resolved within the hour. Two more syncopal episodes occurred overnight. The patient developed left-sided hemiplegia later that day on September 21. CT imaging confirmed a right parietal lobe infarct. Hematological investigations revealed a white blood cell count of 10.8, lymphocytes 12.5%, neutrophils 80.2%, hemoglobin 10.4, MCV 81, platelets 185, and C-reactive protein (CRP) 8.8 mg/dl. Biochemistry investigations showed sodium (Na) 130, potassium (K) 4.5, chlorine (Cl) 91, chromium (Cr) 1.2, blood urea nitrogen (BUN) 29, lactate dehydrogenase (LDH) 1170, aspartate aminotransferase (AST) 736, Alanine transaminase (ALT) 428, Alkaline phosphatase (ALP) 98, total bilirubin 1.3, direct bilirubin 0.3, total protein 6.1, and albumin 3.4. Incidentally, the patient was diagnosed with aspiration pneumonia at the hospital, which was resolved with a course of metronidazole.

**Figure 1:**
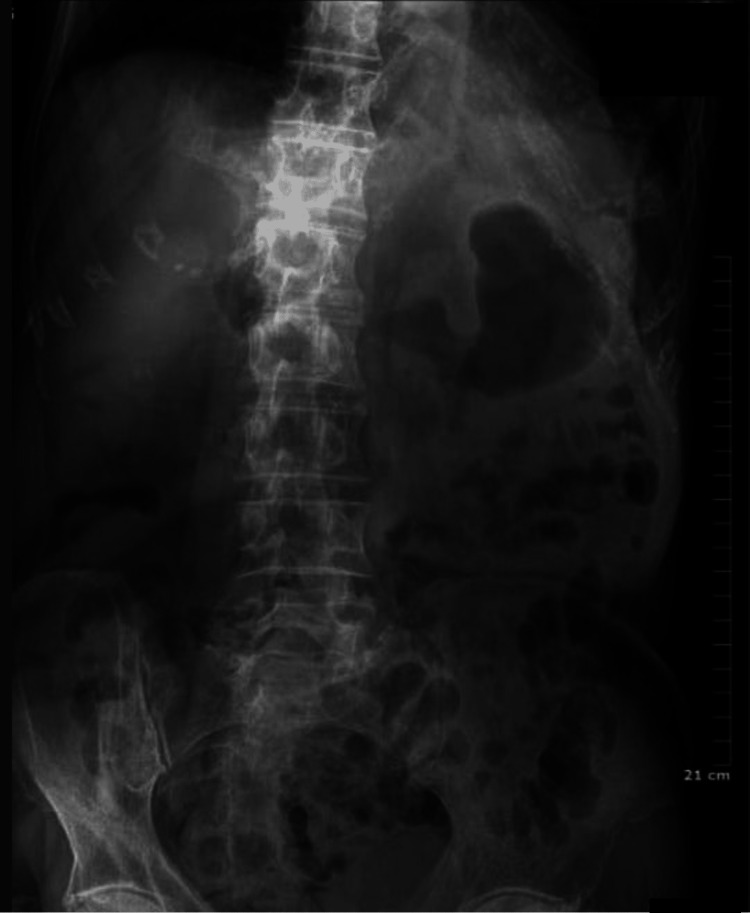
Abdominal X-ray showing fecal matter in colon

Since the stroke, the patient’s oral intake had been poor with intermittent dysphagia. The patient had been constipated since the day of the stroke. A digital rectum exam found no stool in the rectum. A portable X-ray machine was used to perform an abdominal scan at home that displayed stool within the large colon on the left as demonstrated in Figure 1.

Nasogastric intubation was attempted to provide fluids and medications orally but was unsuccessful due to poor patient cooperation. Two bottles of enemas were administered with little relief and prompted the family to admit him to the hospital. His vital signs on admission were as follows: blood pressure 103/65 mmHg, pulse 75 bmp, respiratory rate 18 bpm, oxygen saturation (SpO2) 96%, and random blood glucose 117 mg/dl. A CT scan performed showed fecal loading in the right and left colon with no bowel obstruction, coronary artery and aortic valve calcifications with no cardiomegaly, and features of mild interstitial lung disease at the lung bases. The patient passed soft stool after the CT scan. Additional edemas were given and successfully deloaded the colon. The family requested an echocardiogram and Holter monitor for the patient during his stay. The echocardiogram showed an ejection fraction of 48-50% with the rest of the findings being consistent with the echocardiogram done in 2021. The Holter monitor showed frequent pre-ventricular and pre-atrial contractions as well as a short run of supraventricular ectopy lasting less than two seconds. The patient did not experience any syncope at rest during the Holter monitor test. On October 25, the patient had another syncopal episode with agonal breathing after which the patient died. 

## Discussion

This case represents an atypical presentation of aortic stenosis in which the patient presents with syncope at rest followed by vomiting in the absence of shortness of breath or chest pain. The absence of chest pain is attributable to the patient’s diabetes. A study conducted on aortic stenosis patients with syncope found that only 7.3% of syncope was due to aortic stenosis and that aortic stenosis was only a mild contributing factor in the other cases of syncope [7].

Henceforth, this patient presents evidence to support nonvalvular factors that cause syncope. For instance, this patient’s hyperorality is likely to be secondary to Alzheimer’s disease developed after May. This coincides with the second syncopal episode and the subsequent syncope, which progressively became more frequent. All of these episodes were at rest and almost exclusively occurred between 5 to 7 pm. It can be deduced that hyperorality resulted in the accumulation of food in the bowel throughout the day with maximal food being digested after dinner. The concomitant diversion of blood to the bowel coupled with the limited cardiac output can result in systemic hypotension, and thus syncope. This phenomenon has been described in the literature as postprandial hypotension where it is stated that limited cardiac output, elderly age, and Alzheimer's disease are all risk factors which is consistent with this patient’s history [8]. The variable with the highest sensitivity and specificity for postprandial hypotension was constipation (89.6 %), which is consistent with this patient’s history [9].

Albeit there is evidence for postprandial hypotension-induced syncope, the presence of arrhythmia must also be considered. The patient has moderate mitral regurgitation with atrial dilation, a risk factor for atrial fibrillation. Albeit the Holter monitor was ineffective in detecting a life-threatening arrhythmia, it is possible that the arrhythmia was paroxysmal as the patient did not experience any syncope during the 24 hours the monitor was run. Another possible explanation is that vomiting is associated with a parasympathetic activation which may result in bradycardia and thus trigger syncope in an octogenarian with a degenerative conduction system. However, this patient only developed vomiting approximately 10 minutes after recovery from syncope, which may make the parasympathetic association less likely.

Furthermore, in a study, 70 (16.1%) out of 435 candidates for transaortic valve repair were diagnosed with arrhythmias the day before the procedure, with paroxysmal atrial fibrillation/atrial tachycardia being the most common type [10]. Similarly, in another study, 131 (33.7%) of 389 high-risk patients undergoing transcatheter aortic valve implantation were reported to have atrial fibrillation [11].

This case study aims to establish an association between syncope at rest and hyperorality in the setting of aortic stenosis, which has never been described so far. Furthermore, it aims to spread awareness that the presence of syncope without exertion in aortic stenosis should prompt further investigation for an etiology and should not be immediately attributed to aortic stenosis. This case also highlights an unusual presentation of aortic stenosis where syncope occurs at rest with vomit in the absence of chest pain or shortness of breath. If an underlying etiology can be determined, management can be implemented to limit syncopal episodes and improve the quality of life. For instance, meals can be reduced in postprandial hypotension and antiarrhythmics can help prevent arrhythmias from occurring.

There exists a confounding factor in this study; the patient’s tamsulosin was discontinued on August 24. The patient experienced syncope on August 25 likely due to a high bioavailability still in circulation. However, the next episode occurred on September 17, representing a break in the frequency pattern of every few days. This is likely due to tamsulosin-induced hypovolemia no longer compounding the postprandial hypotension. A limitation of this study was detecting any possible arrhythmia due to the unpredictability of the syncope. The Holter monitor was run for only 24 hours and the patient had no syncope. Repeat Holter testing with longer 72-hour periods would increase the diagnostic power of this measure. Alternatively, the use of an implantable loop recorder can be utilized to provide continuous monitoring of the cardiac rhythm to detect an arrhythmia.

Learning points from this case include maintaining a high index of suspicion for non-valvular pathologies causing syncope at rest in aortic stenosis.

## Conclusions

While valve replacement remains the definitive treatment for aortic stenosis, the disease management regimen should include ruling out arrhythmias and other causes of syncope when it occurs at rest. Patients who develop hyperorality may also develop syncope at rest due to post-prandial hypotension. Being open to diagnosis may lead to early detection and effective treatment.
